# Salivary Microbiome Diversity in Kuwaiti Adolescents with Varied Body Mass Index—A Pilot Study

**DOI:** 10.3390/microorganisms9061222

**Published:** 2021-06-04

**Authors:** Hend Alqaderi, Meganathan P. Ramakodi, Rasheeba Nizam, Sindhu Jacob, Sriraman Devarajan, Muthukrishnan Eaaswarkhanth, Fahd Al-Mulla

**Affiliations:** 1 Department of Oral Health Policy & Epidemiology, Harvard School of Dental Medicine, 188 Longwood Ave, Boston, MA 02115, USA; hend_alqaderi@hsdm.harvard.edu; 2 Department of Genetics and Bioinformatics, Dasman Diabetes Institute, Dasman 15462, Kuwait; rasheeba.iqbal@dasmaninstitute.org (R.N.); sindhu.jacob@dasmaninstitute.org (S.J.); 3 Kuwait School Oral Health Program, Ministry of Health, Salmiya 20005, Kuwait; 4 Hyderabad Zonal Centre, CSIR-IICT Campus, CSIR-National Environmental Engineering Research Institute, Hyderabad 500007, India; pr.meganathan@neeri.res.in; 5 National Dasman Diabetes Biobank, Special Services Facility, Dasman Diabetes Institute, Dasman 15462, Kuwait; sriraman.devarajan@dasmaninstitute.org

**Keywords:** salivary microbiome, adolescents, core microbiome, body mass index, microbial diversity, Kuwait population

## Abstract

The potential role of the salivary microbiome in human diseases has increasingly been explored. The salivary microbiome has been characterized in several global populations, except the Arabian Gulf region. Hence, in this pilot study, we profiled the salivary microbiome of Kuwaiti adolescents with varied body mass indexes (BMI). The analyses of core microbiome composition showed *Firmicutes*, *Bacteroidota*, *Proteobacteria*, *Patescibacteria*, *Fusobacteriota*, *Actinobacteriota*, and *Campylobacterota* as the common phylum found in the Kuwaiti adolescent population. We also illustrated a diverse microbial community among the sampled individuals grouped according to their BMI. Notably, the overweight group was found with a higher number of distinct taxa than other groups. As such, the core microbiome composition was found to be significantly different (*p*-value < 0.001) across different BMI groups. Overall, this pilot investigation outlined the microbial diversity and suggested that changes in salivary microbiome composition in people with obese or overweight BMI might reflect their susceptibility to oral diseases.

## 1. Introduction

Obesity is a chronic health condition determined by an individual’s body mass index (BMI). This complex metabolic disorder is characterized by excessive fat accumulation in the body resulting in adverse health effects. Obesity during childhood and adolescence is a significant risk factor predisposing to life-threatening noncommunicable diseases in adulthood. The escalating global childhood and adolescent obesity rate from 4% in 1975 to over 18% in 2016 [1] is highly alarming. This obesity epidemic is a worldwide growing concern, especially in the oil-rich Arabian Gulf countries, as it increases the risk of comorbid health burdens such as type 2 diabetes, cardiovascular diseases, and some cancer types. Notably, the State of Kuwait tops the Middle East region and leads the United States of America in obesity prevalence at 37% and 23% among adults and adolescents, respectively [1].

In general, the cause of obesity has been associated with environmental, behavioral, and genetic influences. Recently, accumulating scientific evidence has uncovered the involvement of the microbial communities inhabiting the human body as one of the essential factors associated with obesity. Several studies have shown the association of gastrointestinal or gut microbiome alterations with obesity in adults and children. The development of obesity has been attributed to gut microbial dysbiosis, which triggers the host’s inflammatory, immune, and metabolic responses [2,3,4]. A decrease in gut microbiome diversity and richness was observed in people with obesity compared to the normal controls [5]. The proportion of increased *Firmicutes* and decreased *Bacteroidetes* has been determined as a marker for gut microbial dysbiosis in people with obesity [3].

The oral cavity is a potential point of ingress for the microbes into the human body serving as a passage to the gut, mediated by saliva ingestion [6,7]. Inevitably, next to the gut microbiota, the human salivary oral microbiome consists of a highly diverse microbiota, including bacteria, fungi, and viruses [8]. The salivary microbiota’s differential diversity and the occurrence of certain bacterial species have been correlated to common oral diseases such as dental caries and periodontitis and systemic diseases, including diabetes, obesity, and cancer [9]. Of the limited available studies related to obesity, few have demonstrated the association of dysbiotic salivary microbiome with obesity in adults [10,11] and adolescents [12,13,14]. For example, the presence of *Veillonella*, *Haemophilus*, and *Prevotella* was correlated with BMI in a Brazilian adolescent population [13].

Given that the microbiome differs among diseases and varies differently across populations influenced by genetics, lifestyle, food habits, and environment, population-wise microbiome profiling is imperative. It is expected that a dramatic transition in the food habits and lifestyle post-oil discovery should have impacted the diversity of microbial communities in the Arab people inhabiting the Gulf region, which is reflected through the elevated prevalence of modern metabolic diseases. The increasing number of studies related to the microbiome in metabolic health and disease mandates comprehensive microbiome cataloging. Profiling studies applying next-generation sequencing (NGS) technology have not been explored in the Arabian Gulf populations except for a recent large-scale salivary microbiome profiling of the Qatari population [15]. Considering the importance and need, we conducted this NGS-based pilot study to characterize the easily accessible salivary microbiome in the adolescent Kuwaiti population.

## 2. Materials and Methods

### 2.1. Samples

Twenty-three saliva samples of Kuwaiti adolescents used in this study were part of the Kuwait Healthy Lifestyle Study. All information related to sample collection conducted is detailed elsewhere [16]. The study was approved by the Ethical Review Committee of Dasman Diabetes Institute in Kuwait, and the Ministry of Health in Kuwait. Written informed consent from the participants’ parents (or guardians) and consent from the children themselves before study initiation were obtained. The research was conducted following the reporting guidelines (EQUATOR).

### 2.2. DNA Extraction and 16S rRNA Gene Sequencing

DNA extraction was carried out using The PureLink™ Microbiome DNA Purification Kit (Thermofisher, Waltham, MA, USA) and quantification using a Qubit fluorometer (Thermofisher, Waltham, MA, USA), following the manufacturer’s instructions.

A total of 5 ng/μL of microbial DNA was amplified using gene-specific primers that target the bacterial 16S rRNA V3 and V4 regions. The full-length primers with overhang adapter sequences used for the study were, 16S Amplicon PCR Forward Primer: 5′ TCGTCGGCAGCGTCAGATGTGTATAAGAGACAGCC TACGGGNGGCWGCAG and 16S Amplicon PCR Reverse Primer: 5′ GTCTCGTGGGCTCGGAGATGTGTAT AAGAGACAGGACTACHVGGGTATCT AATCC. The PCR reaction consists of 2.5 μL of microbial DNA and 12.5 μL of 2 × KAPA HiFi HotStart Ready Mix PCR mix and 5 μL of 1 μM forward and reverse primers. The thermal amplification program consists of 3 min at 95 °C followed by 25 cycles of 30 s at 95 °C, 30 s at 55 °C and 30 s at 72 °C, and a final extension of 5 min at 72 °C. The resulting PCR product was confirmed on a Bioanalyzer using DNA 1000 chip and purified using AMPure XP beads following the manufacturer’s recommendation (Agilent Technologies, Santa Clara, CA, USA). Subsequently, amplicons were bound to dual indices and Illumina sequencing adapters using the Nextera XT Index Kit (Illumina Inc., San Diego, CA, USA) following the manufacturer’s recommendations. Purified and normalized libraries were multiplexed up to 23 samples and paired-end sequencing was carried out in MiSeq platform (Illumina Inc., San Diego, CA, USA).

### 2.3. Bioinformatics Analyses

The amplicon microbiome data were generated for 23 samples with varying BMI. Initially, the samples with less than 300,000 PE reads were removed from the dataset, which was further preprocessed using the Cutadapt tool [17], and the primers and adapters were removed from the PE reads. Subsequently, the data were analyzed in R version 3.6.3. The data analyses were carried out using the following R packages: DADA2 [18], Biostrings (https://bioconductor.org/packages/Biostrings (accessed on 13 September 2019)), phyloseq [19], microbiome (http://microbiome.github.com/microbiome (accessed on 7 July 2020)), vegan, ggplot2, DECIPHER, phangorn, tidyverse, dplyr, ape, DESeq2 [20], ggrare, reshape, and plyr. Primarily, the tool DADA2 was used for the analyses. The software DADA2 infers amplicon sequence variants (ASVs) which is better for inferring microbiome structure than other algorithms [21]. Thus, this study utilized DADA2 for the analyses. On DADA2, the following criteria were used for preprocessing: the truncation length for R1 and R2 reads were set to be 275 and 225, respectively; the maxEE parameter was set to be 3 for both R1 and R2 reads. After preprocessing, the error rates were evaluated, the reads were dereplicated, the R1 and R2 reads were merged, and finally, the non-chimeric sequences were retained. The fasta sequences from the non-chimeric dataset were retrieved, and the taxonomy for each ASV was assigned to IdTaxa Classifier [22]. For taxonomy assignment, the SILVA_SSU database version r138 (https://www.arb-silva.de/ (accessed on 14 July 2020)) was used, and the confidence threshold of 70% was selected for inferring the taxonomy of ASVs. The ASVs were retained only if the ASV had phylum level information. Further, the dataset was cleaned based on the criteria: the ASV should exist in at least two samples, and the frequency of ASV should be at least 0.001%.

The alpha diversity was calculated using the rarefied dataset. The minimum sequencing depth in the dataset was used as the rarefaction depth. The beta diversity was estimated using the unweighted UniFrac distance. The core microbiome was estimated for each BMI group separately. Briefly, the dataset of each BMI group was separated from the master dataset, and the taxa were filtered using the following criteria: the taxa should exist in at least 50% of samples within the group with an abundance of 0.01%. After retrieving the core microbiome taxa for each BMI group, all the groups’ core microbiome data were merged together for further downstream analyses. The differential abundance of core microbiome taxa between different groups was estimated using the DESeq2 package. The permutational ANOVA (PERMANOVA) with 999 permutations was used to evaluate the differences in the composition of the core microbiome structure across different BMI groups.

## 3. Results

### 3.1. Sample Characteristics

All the individuals included in this study were adolescents aged 17–18 years during sample collection conducted in 2019. The mean age of the 23 Kuwaiti adolescents was 17 (SD 0.6) years. Of the 23 adolescents, most of them were females (91%) and few were males (9%). Based on the BMI-for-age, we grouped the participants into obese (*n* = 8), overweight (*n* = 8), normal (*n* = 5), underweight (*n* = 2) following the CDC Growth Charts (www.cdc.gov/obesity/childhood/defining.html (accessed on 16 March 2020)).

### 3.2. Amplicon Sequencing Data Analyses

The diversity of bacterial taxa in participants’ saliva was studied using the data generated from V3–V4 regions of 16S rRNA. A total number of 8,771,944 PE reads were generated for 23 samples, and the PE reads ranged from 164,134 to 496,543 per sample. While most of the samples yielded, more than 300,000 PE reads, three samples, 11,352, 11363, and 11,421 had less than 300,000 PE reads. As the sample sequencing depth could affect the results [23,24], three samples with lower sequencing depths than other samples were removed from the dataset. The remaining 20 samples were included in the downstream analyses, resulting in a total number of 181, 313–269, 215 non-chimeric sequences per sample. Further, the unique sequences were used for taxonomic analyses.

### 3.3. Alpha Diversity

**Figure 1:**
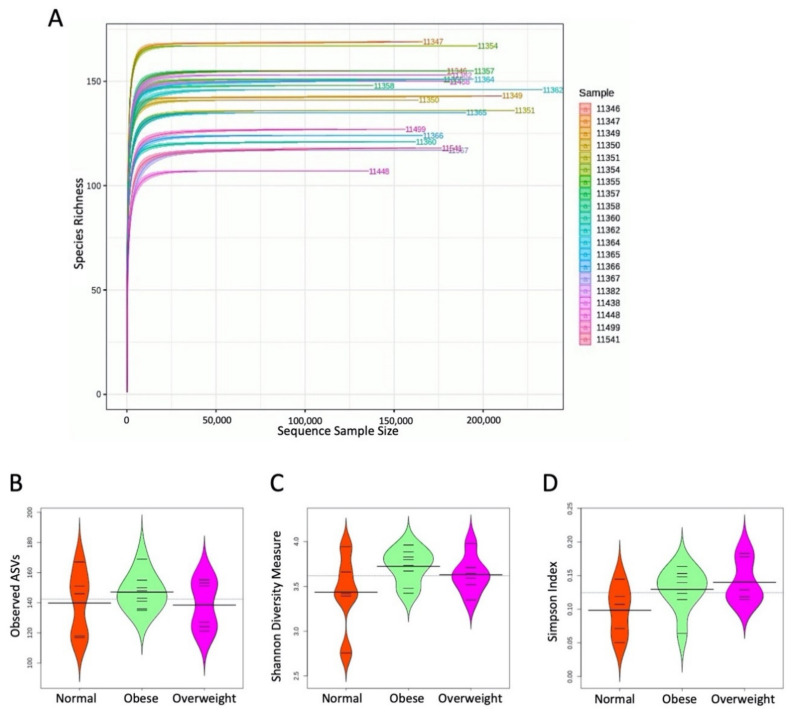
Alpha diversity. (**A**) Rarefaction curve plot showing the sufficient sequencing depth of the samples qualified for diversity analyses; (**B**) distribution of the observed amplicon sequence variants (ASVs); (**C**) Shannon diversity measure; (**D**) Simpson index in each group.

The dataset was rarefied to estimate the alpha diversity indices. The rarefaction curves for the samples are shown in Figure 1A. The results suggest that the sequencing depth generated per sample is sufficient to infer the microbiome community structure. The distribution of observed ASVs, Shannon diversity and Simpson diversity indices were studied in each group, and the results are shown in Figure 1B–D, respectively. Although the alpha diversity indices were found to vary across different groups, the differences were not significant.

### 3.4. Beta Diversity

**Figure 2:**
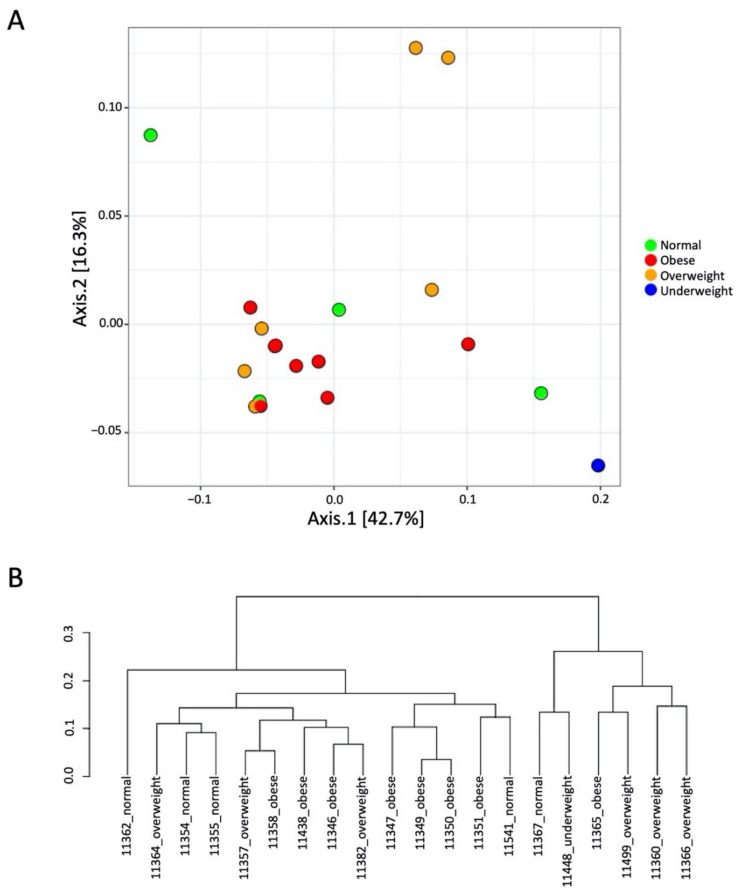
Beta diversity based on unweighted UniFrac measurements. (**A**) Principal coordinate analysis scatter plot showing the distribution of samples based on the microbiome composition; (**B**) dendrogram showing the clustering pattern of samples with varying BMI.

The beta diversity was assessed based on the unweighted UniFrac matrix, which uses phylogenetic information derived from the microbiome composition. The principal coordinate analysis plot and dendrogram based on unweighted UniFrac distance are shown in Figure 2A,B, respectively. The results show that most of the samples were clustered together with other samples of their respective groups. Nonetheless, some samples were found to be clustered with other groups.

### 3.5. Core Microbiome Structure

**Figure 3:**
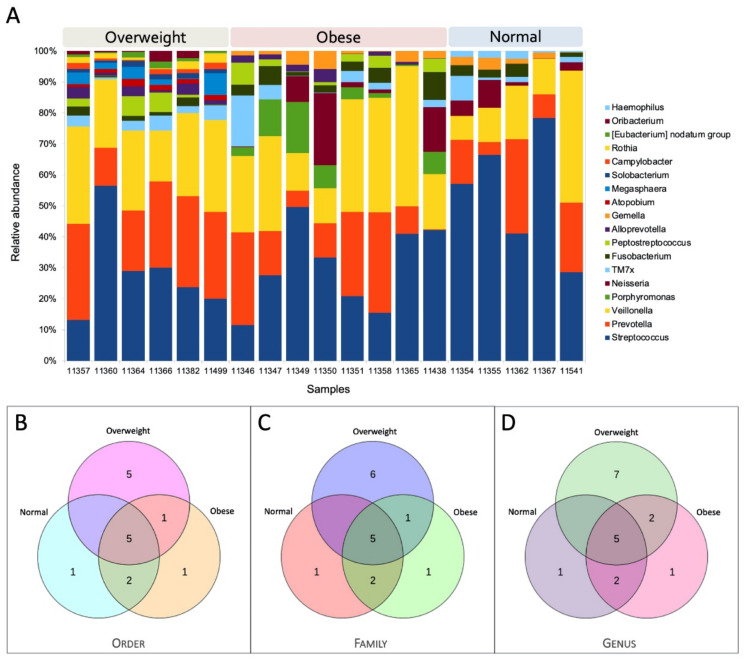
Core microbiome structure. (**A**) Bar plot showing the relative abundance of the core microbiome in each individual; (**B**–**D**) Venn diagrams showing the number of overlapping core microbial taxa based on the microbial taxonomic hierarchy—(**B**) order; (**C**) family; (**D**) genus.

**Figure 4:**
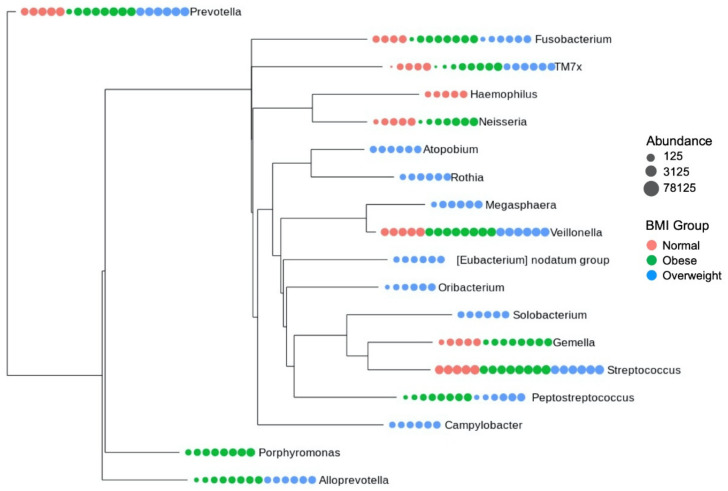
Dendrogram showing the distribution and phylogenetic relationship of the identified core microbial taxa.

The core microbiome structure of each BMI group was analyzed. The analyses show that the composition of core microbiome taxa varies across different groups. The core microbiome composition for each sample is shown in Figure 3A. The comparison of core microbiome taxa indicates that each group uniquely exhibits some taxa (Figure 3B–D and Figure 4). For instance, *Haemophilus* exists only in the normal BMI group, whereas the obese group exclusively had *Porphyromonas* as one of the core microbial taxa. Similarly, *Atopobium*, *Rothia*, *Megasphaera*, *Eubacterium nodatum* group, *Oribacterium*, *Solobacterium*, and *Campylobacter* were found only in the overweight group.

**Figure 5:**
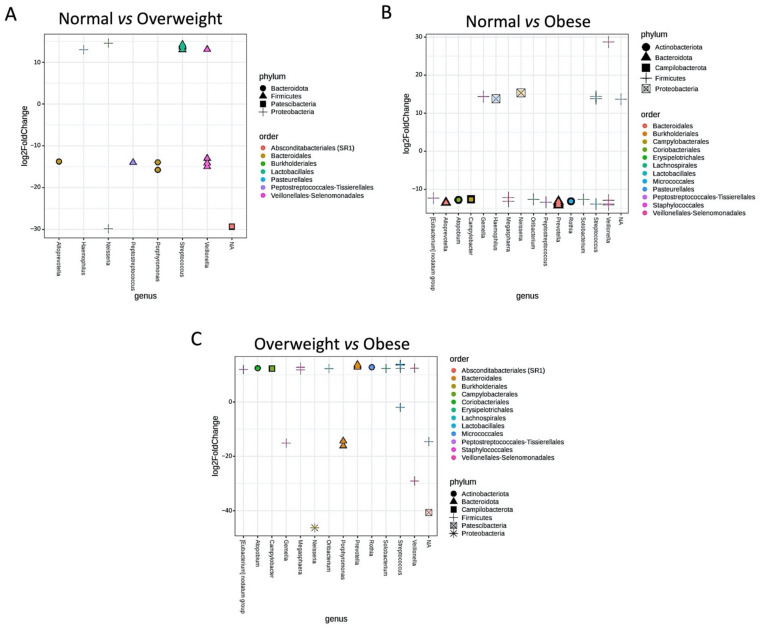
The scatter plots illustrating the differential abundance of core microbial taxa between different BMI groups. (**A**) Normal vs. overweight; (**B**) normal vs. obese; (**C**) overweight vs. obese.

Further, the differential abundance of core microbial taxa between different groups was evaluated based on the log2 fold changes. The normal vs. overweight analyses presented a higher abundance of *Gemella*, *Haemophillus*, and *Neisseria* in the normal group. In contrast, the higher abundance of *Eubacterium notatum* group, *Alloprevotella*, *Atopobium*, *Campylobacter*, *Megasphaera*, *Oribacterium*, *Peptostreptococcus*, *Prevotella*, *Rothia*, and *Solobacterium* were noticed in the overweight group (Figure 5A). The normal vs. obese showed significantly increased abundance of *Haemophilus* and *Streptococcus* genera in the normal group, while an overabundance of *Alloprevotella*, *Peptostreptococcus*, and *Porphyromonas* was exhibited by the obese group (Figure 5B). Finally, overweight vs. obese revealed a significantly increased abundance of *Eubacterium nodatum* group, *Atopobium*, *Campylobacter*, *Megasphaera*, *Prevotella*, *Rothia*, and *Solobacterium* in the overweight group and increased abundance of *Gemella*, *Neisseria*, and *Porphyromonas* in the obese group (Figure 5C). The PERMANOVA analyses showed that the composition of core microbiome across different BMI groups is significantly different (*p*-value < 0.001).

## 4. Discussion

The interest in exploring salivary microbiota has been escalating due to its potential role in human health and disease [9,25]. The immune system has evolved to manage the microorganisms inside and around the human body for nutrition and against possible damage made by those microorganisms [26,27]. Due to the immune reactions such as phagocytosis and xenophagy [28], the human microbiome becomes the prey of human immunity and directly provides essential nutrients to the human body [29], and microbial dysbiosis (the overgrowth of any microbial species at any site of the human body) is the direct cause of the host’s inflammatory and metabolic disorders [30]. Hence, we conducted this NGS-based pilot study to understand the salivary microbial diversity in Kuwaiti adolescents enrolled in a longitudinal study. This preliminary analysis promisingly illustrated a distinct variation of salivary microbiome composition among adolescents grouped based on BMI, i.e., obese, overweight, and normal.

Though the alpha diversity was different across the three BMI groups (Figure 1B–D), the differences were not significant. It is intriguing to note that the obese and overweight groups had an increased number of observed ASVs compared to the normal group, which contrasts with earlier salivary [10,31] and gut [5,32,33,34] microbiome studies which reported lower alpha diversity in obese individuals. However, the association between alpha diversity and obesity varied in different populations. For example, a gut microbiome study [35] illustrated lower alpha diversity in White obese individuals, whereas the obese individuals with Hispanic and African ancestry had higher alpha diversity. Thus, our observation of higher alpha diversity in obese and overweight groups indicates possible localization in the study population. The beta diversity index clustered most of the normal, overweight, and obese individuals with their respective group but some of the individuals were found to be closely related with samples of other BMI groups, which is not surprising, as earlier microbiome studies also noted unexpected clustering patterns of samples with different BMI groups [35].

The analyses of core microbiome composition showed *Firmicutes*, *Bacteroidota*, *Proteobacteria*, *Patescibacteria*, *Fusobacteriota*, *Actinobacteriota*, and *Campylobacterota* as the common phyla found in the Kuwaiti adolescent population. A large-scale salivary microbiome profiling of the Qatari population also demonstrated *Bacteroidetes*, *Firmicutes*, *Actinobacteria*, and *Proteobacteria* as the common taxa [15]. Further analyses of core microbiome taxa showed distinct core microbiome structures in different BMI groups, and the differences were found to be significant. The overweight group was especially found with a higher number of distinct taxa than other groups. Concerning the proportion of microbial populations between groups, obese and normal individuals highly differed in assent with previous studies [10,11,13,31], while the obese and overweight difference was nominal as expected, indicating the plausible progression from overweight to obesity.

In details of the occurrence of a particular bacterial population, *Streptococcus* was found to be common in all the sampled adolescents with varying percentages (12–78%) (Figure 3A); this may indicate the symptoms of dental caries in the analyzed individuals with a likelihood of association between dental caries and obesity, which is unclear at this stage [36]. However, it is to be noted that *Streptococcus* is one of the common bacterial communities found in the human oral microbiome [37,38]. Notably, *Haemophilus* was observed solely in the normal weight Kuwaiti adolescents, which agrees with a previous study that recorded its depletion in obese Chinese individuals [10]. The presence of *Campylobacter* only in overweight individuals is intriguing, as it has been linked with gastrointestinal health [39] and reported to be highly present in obese adolescents of Sweden [40]. Similarly, *Porphyromonas* species was noted exclusively in Kuwaiti adolescents with obese BMI, one of the key periodontal pathogens [41]. In this considerable background, it is plausible that a microbial community could potentially indicate or predispose the obesity status in a Kuwaiti individual. For example, *Prevotella* is one of the core microbiome taxa significantly enriched in individuals with overweight BMI (median relative abundance: normal = 0.142; overweight = 0.278; obese = 0.126) that could be a potential marker to identify individuals at risk of developing obesity as suggested by prior investigations that demonstrated a significant association between BMI and *Prevotella* [31,35]. However, salivary microbiome profiling at a larger scale is required to confirm the validity of the microbial markers.

Our profiling identified the salivary core bacterial microbiota affiliated to the genus *Haemophilus*, *Oribacterium*, *Eubacterium nodatum* group, *Rothia*, *Campylobacter*, *Solobacterium*, *Megasphaera*, *Atopobium*, *Gemella*, *Alloprevotella*, *Peptostreptococcus*, *Fusobacterium*, TM7x, *Neisseria*, *Porphyromonas*, *Veillonella*, *Prevotella*, and *Streptococcus* in Kuwaiti adolescents (Figure 3A). A recent study reported slightly different core salivary bacterial microbiota in Brazilian adolescents, including *Streptococcus*, *Rothia*, *Neisseria*, *Haemophilus*, *Prevotella*, *Actinomyces*, *Porphyromonas*, *Lautropia*, *Granulicatella*, *Gemella*, *Pseudomonas*, *Oribacterium*, and *Fusobacterium* [13]. Likewise, another study suggested a slightly diverse common core salivary microbiome that consists of eight bacterial genera, including *Streptococcus*, *Veillonella*, *Gemella*, *Granulicatella*, *Neisseria*, *Prevotella*, *Rothia*, and *Fusobacterium*, by investigating the oral microbiome of multiethnic adolescent twins and siblings [42]. Collectively, these bacterial populations were found to commonly occur in the human oral microbiome according to the Human Oral Microbiome Database [38,43]. While the previous salivary or gut microbiome studies demonstrated an increased Firmicutes/Bacteroidetes (F/B) ratio in obese individuals [44,45], we noted a decreased F/B ratio in obese and overweight individuals when compared to the normal BMI group. In line with this observation, few prior investigations have also recorded an increased F/B ratio in the normal group compared to obese individuals [46,47]. Magne et al. (2020) reviewed the association between the F/B ratio and obesity and argued the difficulties in accepting the F/B ratio as the hallmark of obesity [48].

In the context of microbial composition specific to different BMI groups, we observed distinct core microbiome structures for each BMI group (Figure 3A). Nonetheless, the core microbiome taxa in obese individuals, observed in earlier studies, are slightly different from this study. Wu et al. (2018) noted the genus *Prevotella*, *Granulicatella*, *Peptostreptococcus*, *Solobacterium*, *Catonella*, and *Mogibacterium* as core salivary microbiome in obese Chinese individuals [10]. Similarly, Liu et al. (2012) recorded the following taxa, *Actinomyces*, *Prevotella*, *Streptococcus*, *Fusobacterium*, *Leptotrichia*, *Corynebacterium*, *Veillonella*, *Rothia*, *Capnocytophaga*, *Selenomonas*, *Treponema*, and TM7 as core microbiome [49]. The reason for observing slightly different core microbiome taxa in our study than earlier studies could be due to the population level differences [50]. Earlier studies have shown a strong association between ethnicity and microbiome composition [51,52,53,54,55,56,57]. Those studies were conducted in various Western and Asian populations to understand the association between core microbiome and BMI, while our study subjects were from Kuwait, one of the Arabian Gulf nations. Our recent studies disclosed the distinct genetic diversity of Kuwait people compared with the Western and Asian populations [58,59]. Thus, the slight difference observed in the core microbiome composition in our study compared to earlier studies could be attributed to the population genetics in addition to the lifestyle and environmental variables. However, further detailed studies are warranted to confirm the core microbiome taxa localized in the Kuwait population.

## 5. Conclusions

This pilot study profiled the salivary microbiome of Kuwaiti adolescents and unraveled its diversity, providing an overview for future studies. Most likely, our observations on microbial diversity may reflect the oral health of the sampled adolescent individuals. This pilot study’s limitation was a small sample size, which could have contributed to the fact that no associations were observed. This study is preliminary and further directed toward the large-scale cataloging of the salivary microbiome in the Kuwait population.

## Data Availability

The data presented in this study are available upon reasonable request from the corresponding author.
